# Aging Kit Mutant Mice Develop Cardiomyopathy

**DOI:** 10.1371/journal.pone.0033407

**Published:** 2012-03-13

**Authors:** Lei Ye, Eric Yang Zhang, Qiang Xiong, Clinton M. Astle, Pengyuan Zhang, Qinglu Li, Arthur H. L. From, David E. Harrison, Jianyi Jay Zhang

**Affiliations:** 1 Leilihei Heart Institute, Department of Medicine, University of Minnesota Medical School, Minneapolis, Minnesota, United States of America; 2 Stem Cell Institute, University of Minnesota, Minneapolis, Minnesota, United States of America; 3 The Jackson Laboratory, Bar Harbor, Maine, United States of America; Northwestern University, United States of America

## Abstract

Both bone marrow (BM) and myocardium contain progenitor cells expressing the c-Kit tyrosine kinase. The aims of this study were to determine the effects of c-Kit mutations on: i. myocardial c-Kit^+^ cells counts and ii. the stability of left ventricular (LV) contractile function and structure during aging. LV structure and contractile function were evaluated (echocardiography) in two groups of Kit mutant (W/Wv and W41/W42) and in wild type (WT) mice at 4 and 12 months of age and the effects of the mutations on LV mass, vascular density and the numbers of proliferating cells were also determined. In 4 month old Kit mutant and WT mice, LV ejection fractions (EF) and LV fractional shortening rates (FS) were comparable. At 12 months of age EF and FS were significantly decreased and LV mass was significantly increased only in W41/W42 mice. Myocardial vascular densities and c-Kit^+^ cell numbers were significantly reduced in both mutant groups when compared to WT hearts. Replacement of mutant BM with WT BM at 4 months of age did not prevent these abnormalities in either mutant group although they were somewhat attenuated in the W/Wv group. Notably BM transplantation did not prevent the development of cardiomyopathy in 12 month W41/W42 mice. The data suggest that decreased numbers and functional capacities of c-Kit^+^ cardiac resident progenitor cells may be the basis of the cardiomyopathy in W41/W42 mice and although defects in mutant BM progenitor cells may prove to be contributory, they are not causal.

## Introduction

The Kit oncogene (Kit), formerly known as c-Kit or W, codes for the c-Kit receptor (a tyrosine kinase). Active c-Kit receptors are essential regulators of hematopoietic stem cell (HSCs) and myeloid progenitor cell functions [1], [2] and are also involved in melanoblastic and gonadal stem cell functions [3]. Kit mutants are generally dominant for white spotting, but recessive for severe effects on hemopoietic precursors and for macrocytic anemia [4]. Anemia is a common manifestation in Kit mutants and it can be corrected by transplantation of normal bone marrow (BM) into mutant animals [5]–[7].

In normal myocardium, cardiac progenitor cells (CPCs) with functionally competent c-Kit receptors appear to play an important role in maintaining cardiomyocyte replacement; moreover, c-Kit^+^ progenitor cells of BM derivation may also be important for the maintenance of cardiac structure and function [8]–[14]. Hence, it is possible that *functionally* significant mutations of c-Kit receptors in cardiac and/or BM c-Kit^+^ cells might reduce the basal rate of cardiomyocyte replacement sufficiently to impact myocardial function and structure as animals age. Hence, we hypothesized that a cardiomyopathy might develop in c-Kit mutant animals as they aged and we examined the stability of LV structure and function with echocardiography over the first year of life (i.e., until ∼middle age) in several groups of mice with Kit mutations (W/Wv and W41/W42) and in wild type (WT) mice.

We report that adverse changes in LV structure (chamber dilatation and hypertrophy) and LV function were present in W41/W42 mice at 12 months of age. These changes were not present in the hearts of WT or W/Wv mutant groups. Further, replacement of mutant BM with WT BM (by transplantation post irradiation) did not prevent the development of cardiomyopathy in W41/W42 mutant mice. These data support the concept that functionally severe mutations of the c-Kit receptor in CPCs can limit the normal cardiomyocyte replacement process sufficiently to induce cardiomyopathy in “middle aged” mice. The data also show that the presence of normal BM cannot compensate for the presence of Kit mutant CPCs in the W41/W42 hearts despite the fact that comparable numbers of normal (i.e. transplanted) BM cells migrated to the heart in both WT and mutant groups. The current report is the first to demonstrate a severe cardiomyopathy develops in W41/W42 Kit mutant mice during the normal aging process.

## Methods

### Animal model

All experimental procedures were approved by the University of Minnesota and the Jackson Laboratory (JAX) Animal Resources Committee.

### Bone marrow transplantation

To determine if repopulating the mutant bone marrow (BM) with normal BM could prevent the aging associated decrease in LV function present in the W41/W42 KIT mutant mice, green fluorescence protein (GFP) labeled BM cells from C57BL/6-Tg (UBC-GFP)30Scha/J (Stock number 004353, Jax Lab, USA) were isolated from femur and tibia, and used as single cell suspensions as described previously [15], [16]. At 4 months of age, recipients were warmed to dilate tail veins (after irradiation) and twenty million GFP marked BM cells were injected into the lateral tail veins with a 30 gauge needle.

### Left ventricular functional assessment using echocardiography

Transthoracic echocardiograms were performed on mice of 4 and 12 months of ages to compare the LV function of Kit mutant and control mice using a Vevo770 Imaging System (VisualSonics, Canada) equipped with a RMV 707B transducer (15–45MH) (VisualSonics Inc, Canada). In addition to conventional two-dimensional images, M-Mode images of the heart in a parasternal short axis view were acquired. Eight consecutive measurements were obtained for left ventricular internal diameters at end-diastole (LVIDed) and end-systole (LVIDes), using the leading edge method recommended by the American Society of Echocardiography. LV end-diastolic (LVEDV) and end-systolic volumes (LVESV) were derived. Left ventricular ejection fraction (EF) and fractional shortening (FS) were calculated as EF% = (LVIDed^2^-LVIDes^2^)/LVIDed^2^*100%; and FS% = (LVIDed-LVIDes)/LVIDed*100%, respectively.

### Immunohistochemistry

Mouse hearts were explanted and fixed in 10% formalin, and then embedded into paraffin and sectioned at 4 µm thickness for immunohistochemistry study. These heart sections were stained for c-Kit (R&D System, USA), GFP (Abcam, USA), Ki67 (Abcam, USA) expression. The c-Kit or GFP positive tissue sections were counter-stained for the expression of α-sarcomeric actin (α-SA) (Sigma Aldrich, USA) to visualize the structure of myocardium. To quantify c-Kit^+^ cells in heart, total numbers of c-Kit^+^/α-SA^−^ cells were counted and data presented as total cells/cm^2^. To quantify proliferating cardiac cells, total numbers of Ki67^+^ nuclei were counted and data presented as Ki67^+^ nuclei/mm^2^. The GFP positive sections were also counter-stained with Ki67 to determine the contribution of GFP^+^ BM to the pool of proliferating cells in the heart. The percentage of Ki67^+^/GFP^+^ over GFP^+^ cells was calculated.

Mouse heart sections were dually stained for von Willebrand factor-8 (vWF-8) (Dako, Denmark) and smooth muscle actin (Sigma Aldrich, USA) expressions to quantify the vascular density.

### Statistical analysis

All data are expressed as mean ± standard deviation. Differences among groups were analyzed by one-way ANOVA, with post hoc comparisons performed using the Bonferroni test. p values less than 0.05 were considered significant.

## Results

### Echocardiographic evaluation of LV function

**Figure 1:**
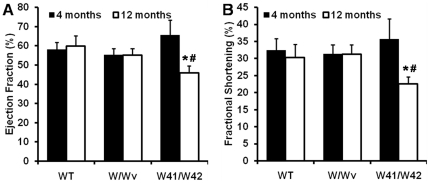
Mouse LV functional studies. (A) ejection fraction, and (B) fractional shortening of mice at both young (4 months) and old (12 months) age. Only W41/W42 mice showed impaired LV function at 12 months of age. *: p<0.05 vs age-matched WT mice. #: p<0.05 vs same group at 4 months of age.

Both left ventricular EF and FS of the mutant and control groups were similar at 4 months of age (Figure 1). However, in 12 month old W41/W42 mice, EF decreased from 66±8% (at 4 months of age) to 46±4 at 12 months of age and FS decreased from 36±6 to 23±2% over the same period (p<0.05 for both). Moreover, the 12 month values in the W41/W42 mice were not only significantly lower than those of WT mice (p<0.05), but also significantly lower than those of the W/Wv mice (p<0.05). On the other hand, no significant differences in LV function were found between 12 month old WT and W/Wv groups (p>0.05).

### Cardiac hypertrophy in W41/W42 mutant mice

**Figure 2:**
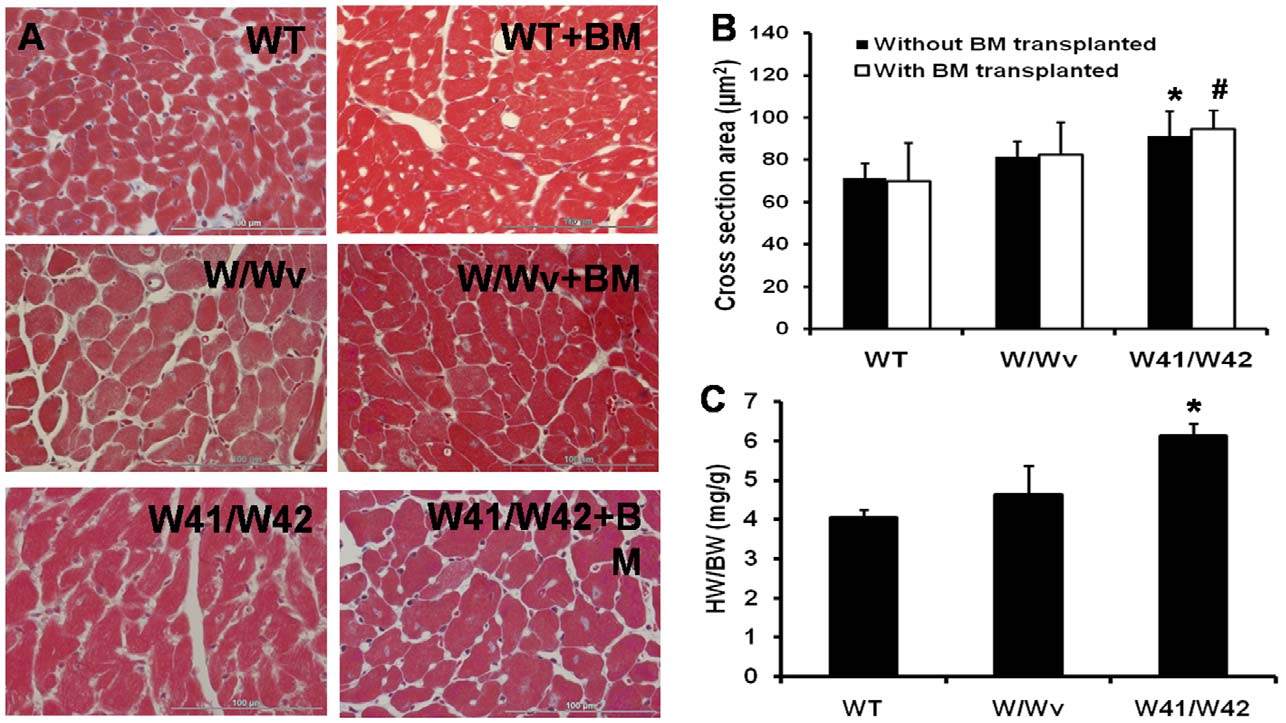
W41/W42 mutant mice had severe cardiac hypertrophy at 12 months of age. (A) Typical H & E staining pictures of mouse heart tissue sections. (B) The mean cross sectional area of cardiomyocytes in W41/W42 mice was significantly increased. (C) Heart weight/body weight (HW/BW) measurements of W41/W42 hearts were also significantly increased. * p<0.05 vs WT mice.

At 12 months of age, cardiomyocyte size of W41/W42 mice was significantly increased as compared with that of WT mice regardless of BM transplantation (Figure 2A–B). Similarly, the heart weight over body weight (HW/BW, mg/g) index was increased by 50% in W41/W42 mice as compared with WT (p<0.05, Figure 2C). Hence, W41/W42 mice had significantly increased LV mass at 12-month of age resulted from cardiomyocyte hypertrophy.

### Reduced myocardial CPCs numbers in all Kit mutant groups

**Figure 3:**
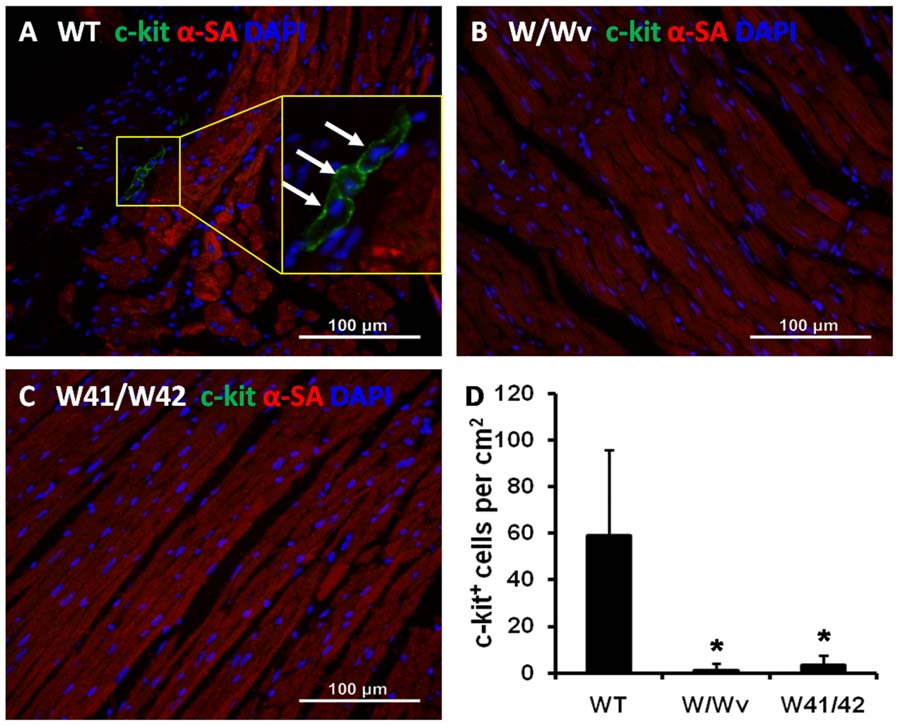
Impaired c-Kit^+^ cardiac progenitor cell pool in Kit mutant mice. Shown in panels (A) to (C) are representative immuno-stained images from hearts of WT, W/Wv, and W41/W42 mice, respectively. Quantification of c-kit^+^ cardiac progenitor cells based on immuno-staining images is shown in panel (D). *: p<0.01 vs WT mice.

Typical immuno-staining of myocardial c-Kit^+^ cells is illustrated in Figure 3A–C. Pronounced reductions of myocardial CPCs numbers were found in both Kit mutant groups as compared to the numbers present in WT mice (p<0.01 for both). WT hearts averaged nearly 58 c-Kit^+^ cells per cm^2^ but very few c-Kit^+^ cells were found in either the Kit mutant group (Figure 3D).

### BM Transplantation did not increase myocardial CPCs numbers in W41/W42 mice

We have previously reported that different type of Kit mutant mice manifested different levels of anemia; importantly, the anemia in all mutant types tested was corrected by transplantation of normal BM following irradiation of the mutant mice [1]. This was not surprising because normal BM maintains hematopoietic functions within the organism and the native BM in the mutants was incapable of adequately performing this function.

**Figure 4:**
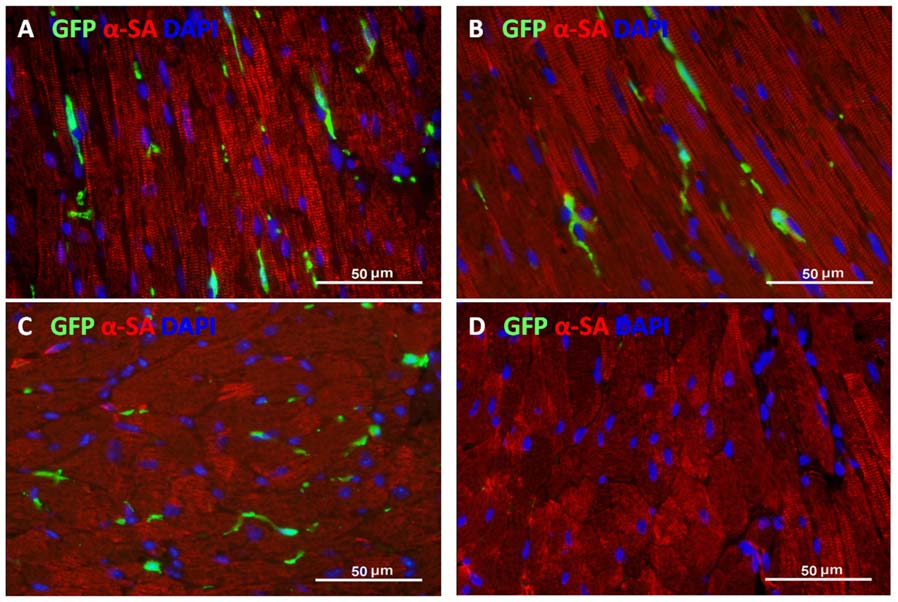
GFP^+^ circulating cells migrate to the hearts of (A) WT, (B) W/Wv and (C) W41/W42 mice. A WT mouse heart without GFP^+^ bone marrow transplantation was shown in panel (D) as a control.

**Figure 5:**
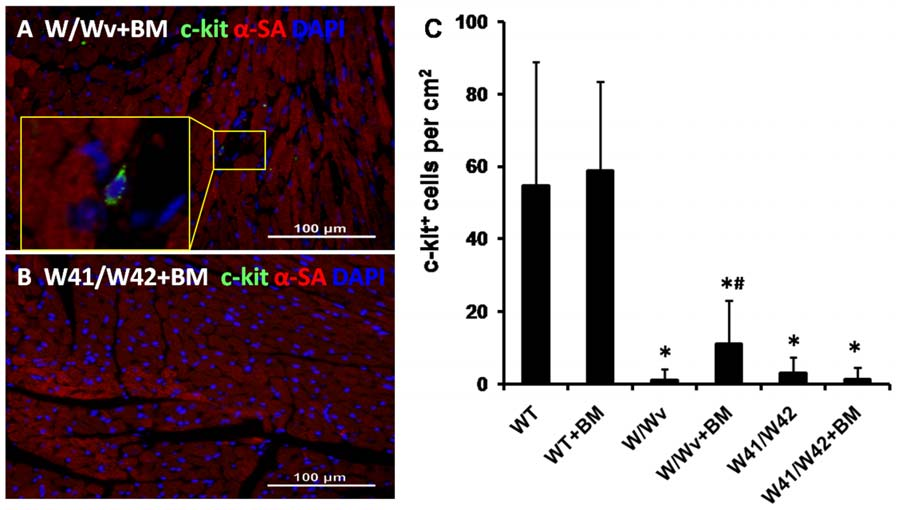
Effects of BM transplantation on c-kit^+^ cardiac progenitor cell pool of Kit mutant mice. Shown in panels (A) and (B) are representative c-kit staining of BM transplanted hearts from W/Wv and W41/W42 mice, respectively. Quantification of c-kit^+^ cell pool based on immuno-staining is shown in panel (C). Bone marrow transplantation did not fully restore the impaired c-Kit^+^ cell pool (*: p<0.05 vs WT). Nevertheless, W/Wv mice (but not W41/W42 mice) modestly increased of c-Kit+ cell numbers in response to BM transplantation (#: p<0.05 vs W/Wv).

BM derived c-Kit^+^ cells are also thought to migrate to the myocardium where they can function similarly to CPCs and participate in cardiac repair [17] and/or activate CPCs (or native cardiomyocytes) to proliferate and differentiate [18]. Hence, we asked whether normal BM cells could perform these functions in the Kit mutant groups. Following BM transplantation, substantial numbers of GFP labeled cells were found in Kit mutant mouse hearts (Figure 4). Moreover, BM transplantation induced a modest, but significant increase of c-Kit^+^ cells in myocardium of W/Wv mice. However, no significant increase of c-Kit^+^ cells occurred in hearts of W41/W42 mice (Figure 5). Cardiac c-Kit^+^ cell counts also did not change in WT mice in response to BM transplantation and the latter remained 10 fold or higher than those present in the BM transplanted Kit mutants (Figure 5). Thus, repopulation of W41/W42 mutant with normal (GFP labeled) cells that migrated from the BM did not increase the numbers of cardiac c-Kit^+^ cells in W41/W42 mice. Hence, the normal BM cells resident in the heart did not stimulate reproduction of cardiac c-Kit^+^ cells in the presence of the most severe form of the mutation. Notably, although there was evidence of a tropic effect of BM derived c-Kit^+^ cells on cardiac endogenous c-Kit^+^ counts in the presence of the less severe W/Wv mutation, even after transplantation these numbers were still markedly lower than those present in normal hearts (i.e., only about 10% of the normal value) (Figure 5).

### Transplantation of normal BM did not prevent the development of cardiomyopathy in W41/W42 mice

**Figure 6:**
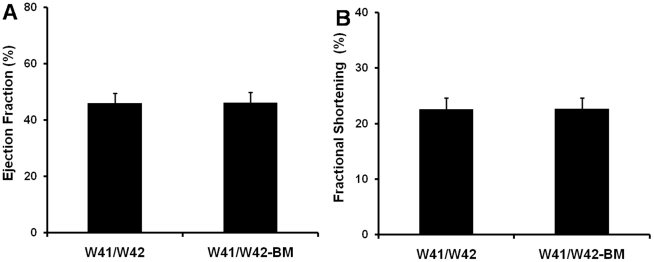
BM transplantation did not prevent LV dysfunction in 12 month old W41/W42 mice. (A) ejection fraction, and (B) fractional shortening.

LV function in hearts of W41/W42 mice at 12 months of age is illustrated in Figure 1. BM transplantation did not prevent the reductions of EF and FS or the development of cardiac hypertrophy inW41/W42 mice at 12 months of age (Figure 1 and 6)

### Transplantation of normal BM did not increase vascularization in hearts of Kit mutant mice

**Figure 7:**
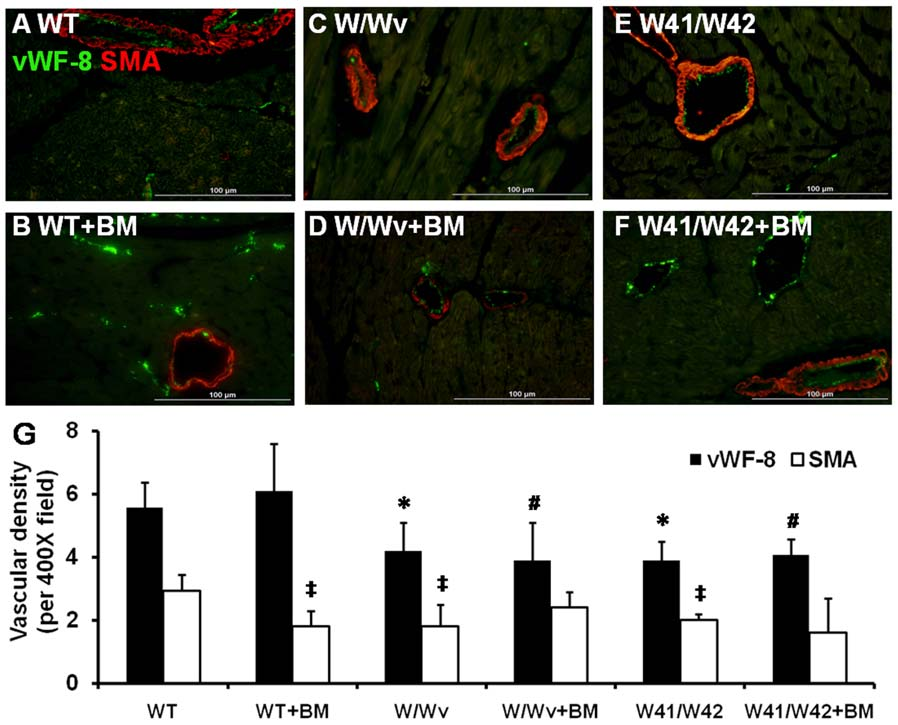
Dual fluorescent immunostaining for vWF-8 and SMA expression. Representative vascular staining pictures of WT (A&B), W/Wv (C&D), and W41/W42 (E&F) myocardium. (G) Quantification of blood vessel number based on vWF-8 and SMA staining indicated that mutant hearts had significantly fewer capillaries and arteioles than did WT mice, BM transplantation did not increase capillary or arteriolar numbers in any group. *: p<0.05 vs vWF-8 of WT; #: p<0.0 vs vWF-8 of WT+BM; ‡: p<0.05 vs SMA of WT.

The Kit mutations were associated with significant reductions of capillary (only vWF-8^+^ vessels) density as compared with that of WT mice even following BM transplantation (Figure 7). Significantly lower arteriole (both vWF-8^+^ and SMA^+^ vessels) density was also found in Kit mutant mice as compared with WT mice. After BM transplantation, a significant (unexplained) reduction of arteriole density was found in WT mice, while arteriolar numbers were not affected in Kit mutant hearts.

### Transplantation of normal BM did not increase cardiac cell proliferation in hearts of Kit mutant mice

**Figure 8:**
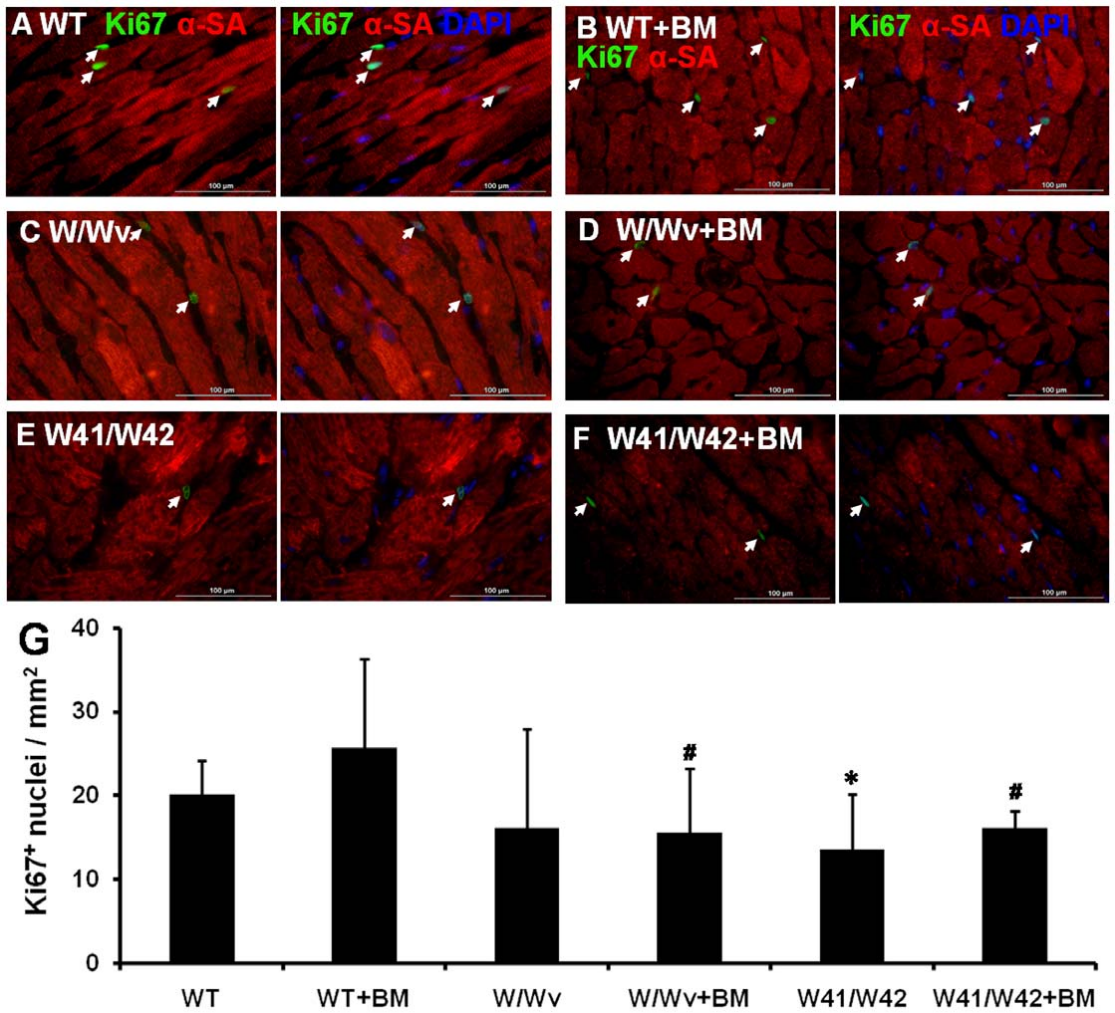
Cardiac cell proliferation based on Ki67 staining. Representative Ki67^+^ staining pictures of WT (A&B), W/Wv (C&D), and W41/W42 (E&F) hearts without or with BM transplantation. Arrows indicate Ki67^+^ nuclei. (G) BM transplantation did not significantly increase Ki67^+^ cell numbers in WT, W/Wv or W41/W42 hearts. *: p<0.05 vs WT, #: p<0.05 vs WT+BM.

**Figure 9:**
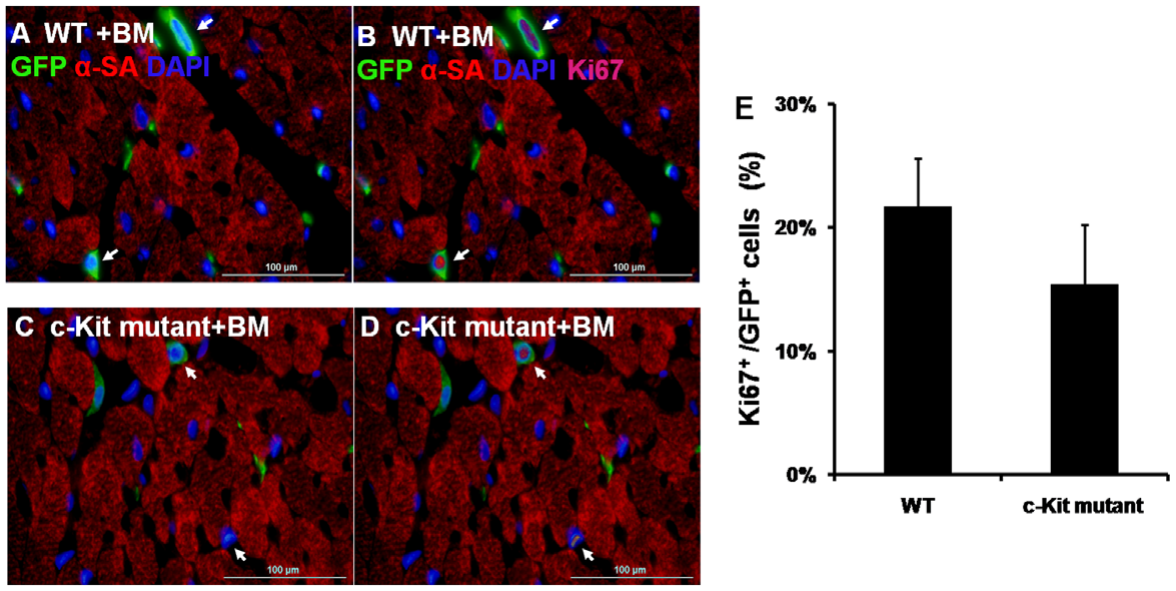
GFP^+^ BM cells (arrows) contribute to Ki67^+^ cardiac cell pool. Representative GFP/Ki67 double-staining pictures of WT (A&B) and c-Kit mutant (C&D) mice after BM transplantation. Arrows indicate GFP^+^/Ki67^+^ nuclei. (E) Quantification of Ki67^+^/GFP^+^ over GFP^+^ cells suggested that 16–22% of BM contributed to the proliferating cell pool in C-Kit mutant and WT mice hearts.

Proliferating cell numbers were similar in W/Wv and WT hearts (Figure 8), however, significantly reduced in W41/W42 hearts. BM transplantation did not significantly increase the proliferating cardiac cell density in WT or mutant hearts. However, proliferating cell numbers were significantly lower in Kit mutant than WT hearts with BM transplantation. A few Ki67^+^ myocyte nuclei were also observed in WT hearts (Figure 8A) while they were rarely seen in Kit mutant hearts. To determine if (migrated) BM cells in the heart might be proliferating, sections were dually stained for GFP and Ki67 (Figure 9). It was found that 15.4±4.8% of GFP BM cells were proliferating in Kit mutant hearts while this value was 21.7±3.9% in WT mouse hearts (p>0.05).

## Discussion

The major finding in this study was that LV hypertrophy, LV chamber dilation and decreased LV performance were present in 12 month old W41/W42 but not W/Wv or WT mice of comparable age. Moreover, myocardial c-Kit^+^ cell counts, vascularization and proliferation rates of transplanted BM derived cells in cardiomyopathic W42/W41 hearts were comparable to those present in normally functioning W/Wv hearts. Importantly, BM transplantation did not prevent the development of cardiomyopathy in W41/W42 mice. Taken together, these data support the hypothesis that the primary cause of the cardiomyopathy in W41/W42 mice are the decreased numbers and functional capacities of CPCs and that functionally defective mutant BM progenitor cells, though possibly contributory to the development of cardiomyopathy, were not causal factor.

There is a large literature describing the roles played by c-Kit receptor bearing progenitor cells in tissue maintenance and regeneration [18]–[21]. Although much of the reported work has been concerned with the hematopoietic system, the roles of resident and/or BM originating c-Kit^+^ progenitor cells in normal cardiomyocyte turnover and regeneration has recently become a major area of investigation and many reports have suggested that resident c-Kit^+^ cardiac progenitor cells are crucial to these processes; it has also been suggested that BM derived c-Kit^+^ progenitor cells themselves may support myocardial regeneration [18]–[21]. Thus *functionally significant* mutations of c-Kit alleles could impact physiological cardiomyocyte turnover rates and, therefore, the maintenance of LV structure and function over the lifespan of the organism. Further, the effects of a decreased cardiomyocyte turnover rate might be cumulative and lead to LV abnormalities later in the life of the organism, the specific time of onset being dependent on the severity of Kit receptor dysfunction.

In this study, we replaced the BM of WT, W/Wv, and W41/W42 mice with GFP labeled but otherwise normal BM to determine if the migration of normal c-Kit^+^ BM cells to the heart was impaired in the mutant hearts, and whether, if this migration was preserved, it would cause endogenous (mutant) cardiac c-Kit^+^ progenitor cells numbers to increase in the mutant groups and, thereby, prevent the development of abnormalities of LV function in the 12-month W41/W42 mice. Notably, despite apparently normal BM cell migratory rates, and mildly increased numbers of c-Kit^+^ progenitor cells in the W/Wv group, c-Kit^+^ progenitor cell numbers were not increased in the W41/42 hearts and LV abnormalities were not prevented.

Collectively, these findings support several conclusions as follows. First, the W41/W42 Kit^+^ cell mutation is associated with the development of LV hypertrophy and dysfunction during normal aging; this finding supports the view that endogenous cardiac c-Kit^+^ cells play an important role in supporting LV structure and function during normal adult life. Second, the BM repopulation study showed unimpaired migration of normal BM originating progenitor cells to the mutant hearts failed to mitigate the LV abnormalities present in 12 months old W41/W42 hearts.

### Consequences of Kit oncogene mutations in cardiac tissues during the aging process

The human heart contains a small number of progenitor cells capable of symmetrical and asymmetrical replication [9], [22]. A variety of such progenitor cells have been identified, including cells expressing Sca-1, c-Kit, islet-1 and side population cells [23]–[25]. Among these, c-Kit has received much attention. c-kit^+^ cardiac progenitor cells have been shown to be self-renewing, clonogenic, and multi-potent, and to exhibit biochemical differentiation into the cardiomyocyte, smooth muscle, and endothelial cell lineages [26]. Moreover, they have been claimed to induce regeneration of functional myocardium by their proliferation and differentiation and/or by activating endogenous cardiac progenitor cells to proliferate and differentiate. In addition, Kit serves as an important developmental cue; not only for cardiomyocyte terminal differentiation but also for regulating the number of cardiac progenitors in the LV [18].

In the current study, each group of Kit mutant mice had normal cardiac function at 4 months of age measured by echocardiography. However, by 12 months of age, severely impaired LV function was found in W41/W42 mice. Notably, the hematological consequences of W42 mutants are known to be more severe that those of the other mutant groups. However, the LV dysfunction probably did not result from the more severe anemia present in W41/W42 mice because this anemia was corrected by the transplantation of normal BM as previously reported [5]. Taken together, these data support the concept that fully functional CPCs are important to the maintenance of normal LV function during aging.

The issue of why LV hypertrophy is present in the older W41/42 hearts is also of interest. It might be hypothesized that the decrease in the rate of cardiomyocyte replacement in that group somehow increases LV stress. Ki67 staining of cardiac tissue showed that although proliferating cell densities were similar between Kit mutant and WT hearts, proliferating cardiomyocytes were only observed in WT hearts. This suggests that in Kit mutant mice CPC-based cardiomyocyte regeneration is reduced and may not be able to match the (required) cardiomyocyte replacement rate. It is also possible that a reduced rate of proliferation of already differentiated cardiomyocytes may be contributory. If present, a reduced cardiomyocyte replacement rate could cause compensatory LV dilation and result in activation of the cardiac renin/angiotensin system which, in turn, could result in cardiomyocyte hypertrophy and dysfunction as well as LV dysfunction [27].

### Transplantation of normal BM fails to rescue LV structure and function in W41/W42 mice

Transplantation of normal BM into a variety of c-Kit mutant types has previously been shown to repopulate hematopoietic stem cell populations and restore populations of red blood cells, etc. [1], [5] As shown in Figure 3, myocardial c-Kit^+^ cell numbers are markedly and comparably decreased in both W/Wv and W41/W42 hearts. However, in the W/Wv group, transplantation of normal BM resulted a moderate, but significant, increase in the level of c-Kit^+^ cells in myocardium (to ∼10% of WT) while in the W41/W42 hearts no increase in the total c-Kit^+^ cell count was observed (Figure 5). These data suggest that the modest increase in the total myocardial c-Kit^+^ cell counts observed in the (transplanted) W/Wv hearts results from expansion of the pre-existing resident c-Kit^+^ cell pool. This expansion may be a consequence of paracrine signaling emanating from the transplanted BM cells that acts on the cardiac endogenous c-Kit^+^ cells. Speculatively, the modest response of the c-Kit^+^ cells in the W/Wv hearts might be interpreted as evidence of superior functional capacity (vs W41/W42 c-Kit^+^ cells) and account for the fact that they did not develop cardiomyopathy by 12 months of age. Moreover, the lack of response of the W41/W42 mutant cardiac c-Kit^+^ cells to paracrine signaling from the transplanted BM cells could indicate lower intrinsic functional capacity than those of their W/Wv counterparts and supports the hypothesis that lower “functional” capacity facilitated the development of the age dependent cardiomyopathy. Collectively, these data are compatible with the concept that resident c-Kit^+^ CPCs are most disabled in W41/W42 mutants.

Kit mutant mice had low capillary and arteriole densities in heart as compared to WT mice and BM transplantation did not increase capillary and arteriole densities in the mutant hearts. This suggests that the mutant micro-vasculature progenitor cells of Kit mutant mice cannot respond to paracrine signaling from the BM derived cells and further, that normal BM derived cells within the myocardium cannot themselves trans-differentiate into micro-vascular cells within the myocardium.

It is also interesting to note that the steady state levels of BM derived cells within the myocardium were comparable in the transplanted WT, W/Wv and W41/W42 groups. Hence, the migratory capacity of the transplanted BM derived cells was presumably normal in all groups and they may have been equally functional in all groups. This reinforces the view that the marrow derived cells themselves, at least in the absence of functional resident cardiac c-Kit^+^ cells, cannot support an adequate rate of cardiomyocyte turnover. Previously, many groups have reported that BM derived c-Kit^+^ cells play a major role in cardiac regenerative processes [11] and the current data suggest that this phenomenon depends on the presence of adequately functioning CPCs.

Our new findings in W41/W42 mutants suggest that this combination of c-kit mutant alleles specifically compromises at least a subset of c-kit functions sufficiently to not only markedly reduce the size of the cardiac resident c-Kit^+^ population but also their physiological functions as the mice age. The present data are compatible with the view that the presence of an at least partially functional c-Kit^+^ endogenous cardiac progenitor population is required for the long term stability of LV structure and function. If it is assumed that the CPCs function that is most markedly attenuated in the W41/W42 mutants is the maintenance of the cardiomyocyte turnover rate, then the present data are in agreement with the recent findings that heart is not a post mitotic organ and that some level of cardiomyocyte turnover via cardiac progenitor cell differentiation does occur during entire life after birth.

In conclusion, our findings in W41/W42 mutants suggest that this combination of c-Kit mutant alleles specifically compromises c-Kit functional capacity sufficiently to: i. markedly reduce the size of the cardiac resident c-Kit^+^ cell population and ii. reduce the capacity of the cardiac resident c-Kit^+^ cell population to support a normal rate of cardiomyocyte replacement. The primary cause of the cardiomyopathy in W41/W42 mice is likely the decreased numbers and functional capacities of *cardiac resident* c-Kit^+^ progenitor cells and not the functional defects mutant BM progenitor cells. This view is supported by the finding that repopulation of the W41/W42 mice with normal BM did not prevent the late development of cardiomyopathy.
